# Cost-effectiveness analysis of first-line penpulimab plus chemotherapy for recurrent or metastatic nasopharyngeal carcinoma in China

**DOI:** 10.3389/fphar.2026.1885531

**Published:** 2026-07-03

**Authors:** Haiyan Xu, Jia Huang, Haoze Bian, Guodong Wang, Feng Xuan

**Affiliations:** 1 Department of Medical Oncology, Zhuji Affiliated Hospital of Wenzhou Medical University, Shaoxing, China; 2 Department of Otolaryngology, Zhuji Affiliated Hospital of Wenzhou Medical University, Shaoxing, China; 3 Department of Pharmacy, Zhuji Affiliated Hospital of Wenzhou Medical University, Shaoxing, China; 4 Department of Radiation Oncology, Zhuji Affiliated Hospital of Wenzhou Medical University, Shaoxing, China

**Keywords:** cost-effectiveness analysis, incremental cost-effectiveness ratio, partitioned survival model, penpulimab, quality-adjusted life-years, recurrent or metastatic nasopharyngeal carcinoma

## Abstract

**Background:**

Recurrent or metastatic nasopharyngeal carcinoma (RM-NPC) remains a major health burden in China. Although penpulimab plus chemotherapy has improved clinical outcomes, its economic value remains uncertain. We evaluated the cost-effectiveness of first-line penpulimab plus chemotherapy versus placebo plus chemotherapy from the perspective of the Chinese healthcare system.

**Methods:**

A partitioned survival model was developed to compare penpulimab plus chemotherapy with chemotherapy alone in patients with RM-NPC. The main outcomes included total costs, life-years (LYs), quality-adjusted life-years (QALYs), incremental cost-effectiveness ratios (ICERs), incremental net monetary benefit (INMB), and incremental net health benefit (INHB). One-way, two-way, probabilistic, price simulation, scenario and subgroup analyses were performed.

**Results:**

Compared with placebo plus chemotherapy, penpulimab plus chemotherapy increased total costs by $ 5,841.38 and generated gains of 0.29 LYs and 0.25 QALYs, yielding an ICER of $23,717.68/QALY. At a willingness-to-pay threshold of $27,906/QALY, the regimen produced a positive INMB of $1,031.53 and a positive INHB of 0.04 QALYs. Sensitivity analyses identified progression-free survival utility, penpulimab cost, and discount rate as the most influential parameters. Only variation in progression-free survival utility increased the ICER above the threshold. The probability of cost-effectiveness was 79.0% at the 2× gross domestic product threshold and 99.2% at the 3× threshold. Penpulimab plus chemotherapy remained cost-effective across all scenario analyses and evaluable subgroups.

**Conclusion:**

From the perspective of the Chinese healthcare system, first-line penpulimab plus chemotherapy is likely to be a cost-effective treatment strategy for patients with RM-NPC compared with placebo plus chemotherapy.

## 1 Introduction

Nasopharyngeal carcinoma (NPC), originating from the nasopharyngeal epithelium, contributes to around 120,434 new cases and 73,482 deaths worldwide annually. (*International Agency for Research on Cancer.*) The primary lesion is often deeply situated and early symptoms are frequently nonspecific, which contributes to delayed diagnosis in routine practice. (Huang et al., 2023). Consequently, a substantial number of patients present with locoregionally advanced or metastatic disease, and 20%–30% of those with advanced disease face treatment failure due to recurrence or metastasis. (Huang et al., 2023; Huang et al., 2026). China bears a disproportionate share of the global NPC burden. According to GLOBOCAN 2022, China accounted for 51,010 new NPC cases and 28,408 deaths, representing one of the heaviest national disease burdens worldwide. (International Agency for Research on Cancer, 2026).

Over the past decade, systemic treatment for recurrent or metastatic nasopharyngeal carcinoma (RM-NPC) has undergone significant evolution. Gemcitabine combined with cisplatin (GP) has been the conventional first-line therapy. (Hong et al., 2021; Zhang et al., 2016). Median overall survival remains disappointing at 20–30 months. (Hong et al., 2021; Huang et al., 2023; Zhang et al., 2016). Recently, immune checkpoint inhibitors (ICIs) have transformed the treatment landscape. Three major phase 3 trials demonstrated the clinical advantage of combining programmed cell death protein 1 (PD-1) blockade with platinum-based chemotherapy. JUPITER-02 demonstrated meaningful gains in both progression-free survival (PFS) and overall survival (OS) when toripalimab was combined with chemotherapy. (Hai-Qiang et al., 2021). In CAPTAIN-1st, camrelizumab plus chemotherapy also demonstrated favorable efficacy, with a PFS hazard ratio (HR) of 0.54 and an OS HR of 0.67 at interim analysis. (Yunpeng et al., 2021). In RATIONALE-309, tislelizumab plus chemotherapy showed sustained benefit with median PFS of 9.6 versus 7.4 months and median OS of 45.3 versus 31.8 months after longer follow-up. (Yunpeng et al., 2023). Collectively, these studies support PD-1 inhibitor–based chemoimmunotherapy as the optimal first-line approach for RM-NPC.

Along with these advances, treatment-related expenditure has also increased. The addition of immunotherapy to first-line treatment can improve survival outcomes, but it also introduces substantial incremental costs associated with drug acquisition, prolonged maintenance therapy, and management of immune-related toxic effects. This issue is particularly relevant in China, where NPC is endemic and where healthcare resource allocation increasingly requires formal pharmacoeconomic evidence. Cost-effectiveness analysis (CEA) therefore serves an important role in informing payer decisions, reimbursement negotiations, and value-based pricing of novel anticancer therapies. (Jing and Liu, 2025).

As a novel anti–PD-1 antibody developed in China, penpulimab has a modified Fc region that eliminates measurable Fc receptor binding and Fc-mediated effector functions, including antibody-dependent cellular cytotoxicity and antibody-dependent cellular phagocytosis. (Dhillon, 2021; Huang et al., 2022; Kinder et al., 2015; Zhang et al., 2018). This structural design distinguishes penpulimab from conventional anti–PD-1 antibodies and may help preserve antitumor immune activity. Since its first approval in China for relapsed or refractory classic Hodgkin lymphoma, penpulimab has been developed across multiple tumor types, including nasopharyngeal carcinoma, non-small-cell lung cancer, hepatocellular carcinoma, and other solid tumors. (Dhillon, 2021). In particular, penpulimab combined with paclitaxel and carboplatin has been approved in China for first-line treatment of locally advanced or metastatic squamous non-small-cell lung cancer, supported by the phase 3 AK105-302 trial. (Zhong et al., 2024). Moreover, anlotinib plus penpulimab has shown efficacy as first-line therapy for advanced hepatocellular carcinoma in the phase 3 ALTN-AK105-III-02/APOLLO study. (Zhou et al., 2024). In RM-NPC, penpulimab plus gemcitabine-platinum significantly improved PFS compared with placebo plus chemotherapy in a randomized phase 3 trial. (Huang et al., 2026). Consequently, penpulimab has received first-line approval for RM-NPC in China and FDA clearance in 2025 for use with chemotherapy. Although pharmacoeconomic analyses of several similar immunotherapy regimens have previously been conducted in RM-NPC, (Kun et al., 2022; Lian et al., 2024; Tang et al., 2024), penpulimab-specific economic evidence remains lacking. In addition, the cost-effectiveness of PD-1 inhibitors depends on regimen-specific survival benefit, treatment duration, drug price, toxicity profile, utility assumptions, and post-progression treatment patterns. Evidence from other domestic PD-1 inhibitors cannot be directly extrapolated to penpulimab.

To address this knowledge gap, we evaluated the cost-effectiveness of first-line penpulimab plus chemotherapy compared with chemotherapy alone for patients with RM-NPC from the perspective of the Chinese healthcare system, providing penpulimab-specific economic evidence to inform reimbursement and clinical decision-making in China.

## 2 Methods

### 2.1 Study design

A cost-effectiveness evaluation was performed to compare first-line penpulimab plus chemotherapy with placebo plus chemotherapy for RM-NPC in China. The analysis followed the perspective of the Chinese healthcare system and complied with the 2022 Consolidated Health Economic Evaluation Reporting Standards (CHEERS) 2022 reporting standards (Supplementary Table S1) (Husereau et al., 2022).

**FIGURE 1:**
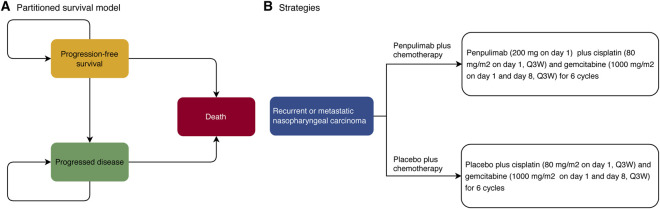
Partitioned survival model consisting of three health states. **(A)** Partitioned survival model overview. **(B)** Treatment strategies for recurrent or metastatic nasopharyngeal carcinoma.

A three-state partitioned survival model [PFS, progressive disease (PD), death] was established to simulate RM-NPC outcomes (Figure 1A). Patients started in PFS and could remain progression-free, progress, or die during each 21-day cycle. PFS and OS curves determined the proportions in PFS and alive, respectively, with PD calculated as the difference between OS and PFS. Outcomes included total costs, life-years (LYs), quality-adjusted life-years (QALYs), and incremental cost-effectiveness ratios (ICERs). Incremental net health benefit (INHB) and incremental net monetary benefit (INMB) were also calculated using the following equations: INHB(λ) = ΔE − ΔC/λ and INMB(λ) = ΔE × λ − ΔC, where ΔC represents the difference in total costs between treatment groups, ΔE represents the difference in QALYs, and λ denotes the willingness-to-pay (WTP) threshold. A lifetime horizon was used since >99% of patients died by model completion. Following the 2025 China Guidelines for Pharmacoeconomic Evaluations, both costs and outcomes were discounted at 4.5% annually, with a WTP threshold of $27,906 per QALY, corresponding to twice China’s gross domestic product (GDP) *per capita*. (Jing and Liu, 2025; Su et al., 2025; Wu and Liu, 2026).

### 2.2 Clinical data, patients, and treatments

Data on patients, treatments, survival, and safety were sourced from a randomized, double-blind phase 3 trial comparing penpulimab plus chemotherapy to placebo plus chemotherapy as first-line therapy for RM-NPC (NCT04974398). (Huang et al., 2026). The study enrolled 291 patients, randomized 1:1 to penpulimab (n = 144) or placebo (n = 147), with both arms receiving chemotherapy every 3 weeks. (Figure 1B). Chemotherapy in the trial consisted of GP-based treatment. After six cycles of combination therapy, patients received maintenance penpulimab or placebo according to the original trial protocol. Penpulimab or placebo maintenance therapy was continued until disease progression, unacceptable toxicity, death, withdrawal, or a maximum duration of 2 years, whichever occurred first. Accordingly, penpulimab acquisition costs were accrued only for patients who remained progression-free and within the 2-year maximum treatment period. The modeled population was consistent with the trial population. For drug dose calculations, all patients were assumed to be aged 51 years and to have a body surface area of 1.72 m^2^ (Chen et al., 2019; Huang et al., 2026; She et al., 2022; Zhu et al., 2022).

### 2.3 Survival data processing and extrapolation

**FIGURE 2:**
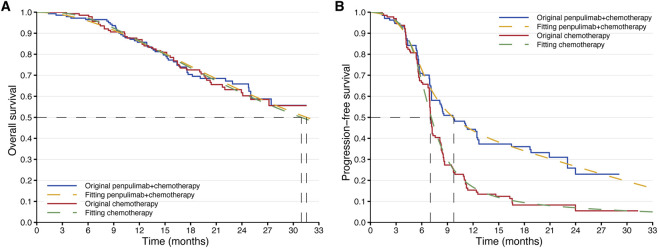
Comparison of fitted and reconstructed survival curves for penpulimab plus chemotherapy versus chemotherapy. **(A)** Overall survival. **(B)** Progression-free survival. This figure assesses the face validity of the partitioned survival model by comparing the reconstructed Kaplan–Meier curves with the survival curves generated from the final selected models.

Due to the lack of publicly available patient-level data, OS and PFS were extracted from the published phase 3 Kaplan-Meier (KM) curves using WebPlotDigitizer (v4.7). (WebPlotDigitizer, 2025). The data were then reconstructed into pseudo-individual patient data via the Guyot algorithm. (Guyot et al., 2012). The reconstructed KM curves are shown in Supplementary Figure S1. Subsequent analyses were conducted in R (v4.4.1) with the flexsurv and survHE packages. (Baio, 2020; Jackson, 2016). OS and PFS were modeled separately for each treatment arm, without assuming a fixed HR relative to the comparator. Given the possibility of time-varying treatment effects with anti-PD-1 immunotherapy, a broad range of conventional and flexible parametric survival functions was assessed, including exponential, Gamma, Gompertz, Weibull, log-logistic, log-normal, generalized Gamma, Royston–Parmar spline, restricted cubic spline, and fractional polynomial models. (Luo et al., 2026). The most appropriate model was identified based on the Akaike information criterion (AIC) and graphical comparisons among the fitted curves, reconstructed KM data, and published KM curves. Models with lower AIC values and better visual agreement were preferred. (Luo et al., 2026). As follow-up data were limited to the duration of the clinical trial, the selected survival functions were extended beyond the observed period to project OS and PFS across the entire analytic horizon and to derive the proportion of patients in each health state within the partitioned survival framework. Detailed fitting outcomes are provided in Figure 2, Supplementary Tables S2–S3 and Supplementary Figures S2–S5. These selected models were then employed to project long-term survival and estimate health-state occupancy over the model horizon.

### 2.4 Costs and utilities

The model included only direct medical costs, encompassing drug acquisition, administration, (Zhu et al., 2022), laboratory tests, (She et al., 2025), tumor imaging, (Zhu et al., 2022), management of adverse events (AEs), subsequent treatment, best supportive care, (Zhu et al., 2022), and end-of-life care. (Pei et al., 2023). Model inputs are summarized in Table 1. Drug acquisition costs were obtained from Yaozhi, (Yao, 2026), a pharmaceutical data platform in China, and the median market price for each drug was used in the base-case analysis to reduce potential bias from single-hospital price estimates. Subsequent treatment costs after disease progression were incorporated into the base-case analysis. The proportions of patients receiving subsequent anti-cancer therapies were extracted from the pivotal phase 3 trial. (Huang et al., 2026). Because detailed regimen-level information was not reported, representative subsequent treatment regimens were selected according to the Chinese Society of Clinical Oncology guidelines for NPC and current Chinese clinical practice (Supplementary Table S4). Other non-drug direct medical costs were derived from previously published studies. All costs were adjusted to 2025 values using the China Consumer Price Index and converted to US dollars at an exchange rate of $1 = 7.14 CNY. In the base-case analysis, only grade ≥3 AEs with >5% incidence were included as one-time costs in the first cycle. (Pei et al., 2023; Qiu et al., 2025). Health outcomes were estimated as QALYs, with utility values ranging from 0, representing death, to 1, representing perfect health. Because EQ-5D or other preference-based quality-of-life data were not reported in the pivotal phase 3 trial, health-state utilities were derived from published pharmacoeconomic studies in RM-NPC. In the base-case analysis, the utility values were 0.76 for PFS and 0.57 for PD. (She et al., 2025; She et al., 2022; Xu et al., 2024; Yen et al., 2009). These utility values were varied over predefined ranges in deterministic sensitivity analyses and assigned beta distributions in the probabilistic sensitivity analysis.

**TABLE 1:** Summary of model inputs and ranges.

Parameters	Baseline	Minimum	Maximum	Distribution	References
Survival data distribution parameter input					
RP-odds-1 OS survival model of penpulimab plus chemotherapy group	gamma0 = −1.82542; gamma1 = 1.88786; knot = 0			Fixed	Huang et al. (2026); Luo et al. (2026)
RP-normal-1 OS survival model of placebo plus chemotherapy group	gamma0 = −1.09821; gamma1 = 1.15973; knot = 0			Fixed	Huang et al. (2026); Luo et al. (2026)
RCS2 PFS survival model of penpulimab plus chemotherapy group	(Intercept) = −0.57309; s (Time).1 = 0.69465; s (Time).2 = −1.57254; s (Time).3 = 0.62932			Fixed	Huang et al. (2026); Luo et al. (2026)
RP-odds-2 PFS survival model of placebo plus chemotherapy group	gamma0 = 0.01623; gamma1 = 2.41020; gamma2 = −4.09688; gamma3 = 4.50674; knot = 2			Fixed	Huang et al. (2026); Luo et al. (2026)
Risk for main AEs in penpulimab plus chemotherapy group					
Anemia	0.45	0.36	0.54	Beta	Huang et al. (2026)
White blood cell count decreased	0.54	0.43	0.65	Beta	Huang et al. (2026)
Neutrophil count decreased	0.56	0.45	0.67	Beta	Huang et al. (2026)
Platelet count decreased	0.30	0.24	0.36	Beta	Huang et al. (2026)
Hyponatraemia	0.05	0.04	0.07	Beta	Huang et al. (2026)
Hypokalemia	0.06	0.05	0.07	Beta	Huang et al. (2026)
Risk for main AEs in placebo plus chemotherapy group					
Anemia	0.39	0.31	0.46	Beta	Huang et al. (2026)
White blood cell count decreased	0.55	0.44	0.66	Beta	Huang et al. (2026)
Neutrophil count decreased	0.62	0.50	0.74	Beta	Huang et al. (2026)
Platelet count decreased	0.36	0.29	0.43	Beta	Huang et al. (2026)
Utility and disutility	​	​	​	​	​
Utility of PFS	0.76	0.61	0.91	Beta	She et al. (2025); She et al. (2022); Xu et al. (2024); Yen et al. (2009)
Utility of PD	0.57	0.46	0.68	Beta	She et al. (2025); She et al. (2022); Xu et al. (2024); Yen et al. (2009)
Disutility of anemia	0.073	0.0584	0.07008	Beta	Tang et al. (2024)
Disutility of white blood cell count decreased	0.090	0.072	0.0864	Beta	Tang et al. (2024)
Disutility of neutrophil count decreased	0.090	0.072	0.0864	Beta	Tang et al. (2024)
Disutility of platelet count decreased	0.200	0.160	0.192	Beta	Tang et al. (2024)
Disutility of hyponatremia	0.080	0.064	0.0768	Beta	Li et al. (2020); Zhao et al. (2023)
Disutility of hypokalemia	0.008	0.0064	0.00768	Beta	Liu et al. (2023); Zhu et al. (2025)
Drug cost ($)	​	​	​	​	​
Penpulimab per mg	1.2611	1.0089	1.5133	Gamma	Yao (2026)
Cisplatin per mg	0.0887	0.0710	0.1064	Gamma	Yao (2026)
Gemcitabine per mg	0.0098	0.0078	0.0117	Gamma	Yao (2026)
Expenditures on main AEs ($)	​	​	​	​	​
Anemia	539.15	431.32	646.98	Gamma	Pei et al. (2023)
White blood cell count decreased	22.30	17.84	26.77	Gamma	Qiu et al. (2025)
Neutrophil count decreased	22.30	17.84	26.77	Gamma	Qiu et al. (2025)
Platelet count decreased	1554.49	1243.59	1865.39	Gamma	Qiu et al. (2025)
Hyponatraemia	9.48	7.58	11.38	Gamma	Qiu et al. (2025)
Hypokalemia	6.38	5.10	7.65	Gamma	Qiu et al. (2025)
Cost of the laboratory test	74.85	59.88	89.82	Gamma	She et al. (2025)
Cost of tumor imaging	232.02	185.62	278.43	Gamma	Zhu et al. (2022)
Cost of administration	106.62	85.30	127.95	Gamma	Zhu et al. (2022)
Cost of best supportive care	158.23	126.58	189.88	Gamma	Zhu et al. (2022)
Cost of terminal care in end-of-life	1840.33	1472.27	2208.40	Gamma	Pei et al. (2023)
Other parameters	​	​	​	​	​
Body area surface/m^2^	1.72	1.38	2.06	Normal	Chen et al. (2019); She et al. (2022); Zhu et al. (2022)
Discount rate (%)	4.5	0	5	Beta	Jing and Liu (2025); Wu and Liu (2026)

Abbreviation: AE, adverse event; PFS, progression-free survival; PD, progressive disease.

### 2.5 Sensitivity analyses

Robustness of the base-case results and parameter uncertainty were examined using one-way, two-way, and probabilistic sensitivity analyses (PSA). One-way analysis varied each parameter individually while keeping others constant, applying ±20% variation when published ranges were unavailable. Results were displayed as tornado diagrams to identify parameters with the greatest influence on the ICER. Two-way sensitivity analysis simultaneously varied two key parameters to assess their combined effect and determine threshold combinations where penpulimab plus chemotherapy might be cost-effective. PSA involved 1,000 Monte Carlo simulations, sampling costs from gamma distributions and utilities and AE probabilities from beta distributions, with results shown as incremental cost-effectiveness scatterplots and cost-effectiveness acceptability curves (CEACs).

### 2.6 Price simulation analysis

To further explore the economic consequences of alternative pricing strategies, a price simulation analysis was conducted for penpulimab. Because national reimbursement negotiations in China may substantially reduce the prices of anticancer drugs, particularly when new indications are approved, we assessed the impact of varying penpulimab prices on total costs and ICERs. The most recent available penpulimab price was used in the base-case analysis, and additional simulations were performed across a range of hypothetical price reductions to estimate the potential effect of future pricing negotiations on cost-effectiveness.

### 2.7 Scenario analyses

To further examine the robustness of the base-case findings, three scenario analyses were conducted. As recommended by the *China Guidelines for* Pharmacoeconomic Evaluations 2025, alternative discounting assumptions were explored. (Jing and Liu, 2025; Su et al., 2025; Wu and Liu, 2026). Scenario 1 applied a discount rate of 0% to both costs and health outcomes, whereas Scenario 2 applied a discount rate of 5%. In addition, to address the potential underestimation of immunotherapy-related toxicity costs, Scenario 3 incorporated low-grade but frequent immune-related AEs that were not included in the base-case analysis. Detailed incidence data, cost inputs, and modeling approaches for these AEs are provided in the Supplementary Table S5.

### 2.8 Subgroup analysis

Subgroup analyses were conducted to evaluate the cost-effectiveness of penpulimab plus chemotherapy across patient subgroups identified in the trial. Subgroup-specific HRs for OS and PFS comparing penpulimab plus chemotherapy with placebo plus chemotherapy were extracted for age, sex, programmed cell death ligand 1 (PD-L1) expression level, Eastern Cooperative Oncology Group (ECOG) performance status, liver metastases, investigator’s choice of chemotherapy, smoking status, disease stage at study entry, Epstein-Barr virus (EBV) DNA level, and geographic region. Because subgroup-specific Kaplan–Meier curves, patient-level survival data, and placebo-group subgroup survival data were not available, the overall population’s survival data from the chemotherapy group were used as the baseline survival functions. Subgroup-specific survival outcomes were then estimated by applying the corresponding subgroup HRs to these baseline survival functions within a proportional hazards framework. (Ding et al., 2021; Wang et al., 2025; Ying-Tao et al., 2023). This approach provided approximate estimates of subgroup-specific OS and PFS for the economic evaluation.

All data processing, survival reconstruction, model analyses, and visualizations were conducted using R (v4.4.1) and Microsoft Excel.

## 3 Results

### 3.1 Base-case analysis

Compared with placebo plus chemotherapy, penpulimab plus chemotherapy increased total costs from $12,513.25 to $18,354.63, resulting in an incremental cost of $5,841.38 (Table 2). However, penpulimab plus chemotherapy also improved effectiveness, with LYs increasing from 3.08 to 3.37 and QALYs increasing from 1.92 to 2.17, corresponding to incremental gains of 0.29 LYs and 0.25 QALYs, respectively. Accordingly, the ICER for penpulimab plus chemotherapy was $20,239.32/LY gained and $23,717.68/QALY gained. At the prespecified WTP threshold of $27,906/QALY, penpulimab plus chemotherapy was associated with a positive INMB of $1,031.53 and a positive INHB of 0.04 QALYs. These findings indicate that penpulimab plus chemotherapy is likely to be a cost-effective first-line treatment strategy relative to placebo plus chemotherapy in the Chinese setting.

**TABLE 2:** Results of the base-case analysis and the scenario analysis.

Strategies	Cost ($)	Overall LYs	Overall QALYs	ICER/LY	ICER/QALY	INMB ($)	INHB (QALY)
Base-case analysis results							
Placebo plus chemotherapy	12,513.25	3.08	1.92	NA	NA	NA	NA
Penpulimab plus chemotherapy	18,354.63	3.37	2.17	20,239.32	23,717.68	1,031.53	0.04
Scenario 1: Discount rate 0%							
Placebo plus chemotherapy	13,596.84	3.40	2.12	NA	NA	NA	NA
Penpulimab plus chemotherapy	19,632.01	3.80	2.43	14,961.00	19,574.62	2,568.70	0.09
Scenario 2: Discount rate 5%							
Placebo plus chemotherapy	12,411.51	3.05	1.91	NA	NA	NA	NA
Penpulimab plus chemotherapy	18,233.06	3.33	2.15	20,879.10	24,166.56	900.80	0.03
Scenario 3: Including low-grade but frequent immune-related AEs							
Placebo plus chemotherapy	12,513.25	3.08	1.92	NA	NA	NA	NA
Penpulimab plus chemotherapy	18,361.65	3.37	2.17	20,263.65	23,922.75	973.79	0.03

Abbreviation: LY, life-year; QALY, quality-adjusted life year; ICER, incremental cost-effectiveness ratio; INMB, incremental net monetary benefits; INHB, incremental net health benefits.

### 3.2 Sensitivity analyses

**FIGURE 3:**
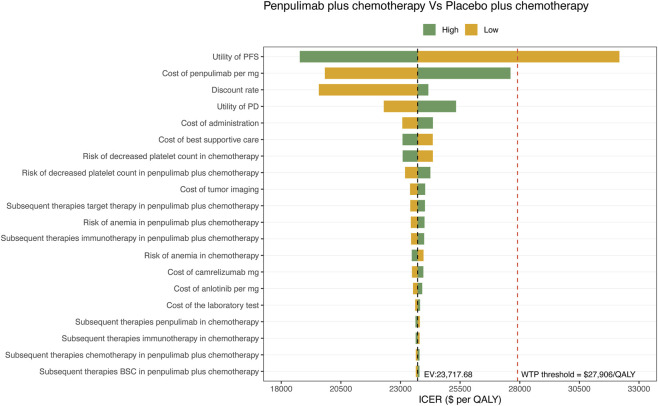
Tornado diagram for one-way sensitivity analysis. Abbreviation: PFS, progression-free survival; PD, progressive disease; ICER, incremental cost-effectiveness ratio; QALY, quality-adjusted life year.

Figure 3 presents the one-way sensitivity analysis. Model outputs were most sensitive to PFS utility, then penpulimab cost per mg, discount rate, and PD utility. When the utility of PFS was varied across its range, the ICER changed from $18,780.05/QALY to $32,177.83/QALY. Corresponding ICER ranges were from $19,825.90 to 27,609.46/QALY for the cost of penpulimab per mg, from $19,574.62 to 24,166.56/QALY for discount rate, and from $22,299.50 to 25,328.49/QALY for PD utility. All other parameters had only modest effects on outcomes. Notably, at the WTP threshold of $27,906/QALY, only variation in the utility of PFS caused the ICER to exceed the threshold, suggesting that the base-case conclusion was generally robust but remained sensitive to uncertainty in PFS utility estimates.

Two-way sensitivity analysis was performed to examine the joint effects of PFS utility and penpulimab cost per mg, which were the two most impactful parameters in the one-way analysis (Table 3). When the utility of PFS was fixed at its lower bound (0.61), the ICER increased from $26,897.84/QALY at the lower-bound drug cost to $32,177.83/QALY at the base-case cost and $37,457.82/QALY at the upper-bound cost. Under the latter two scenarios, the INMB became negative (–$775.48 and –$1,733.98, respectively), indicating loss of cost-effectiveness. By contrast, when the utility of PFS was set at the base value (0.76) or upper bound (0.91), all ICER estimates remained below the WTP threshold despite variation in penpulimab cost, and all corresponding INMB values remained positive.

**TABLE 3:** Two-way sensitivity analysis results of two key parameters.

Results	Parameters	Cost of penpulimab per mg		
	Utility of PFS	Lower bound (1.009)	Base value (1.2611)	Upper bound (1.5133)
ICER/QALY	Lower bound (0.61)	26,897.84	32,177.83	37,457.82
	Base value (0.76)	19,825.90	23,717.68	27,609.46
	Upper bound (0.91)	15,698.47	18,780.05	21,861.63
INMB ($)	Lower bound (0.61)	183.02	−775.48	−1,733.98
	Base value (0.76)	1,990.03	1,031.53	73.04
	Upper bound (0.91)	3,797.05	2,838.55	1,880.05

Abbreviation: PFS, progression-free survival; QALY, quality-adjusted life year; ICER, incremental cost-effectiveness ratio; INMB, incremental net monetary benefits.

**FIGURE 4:**
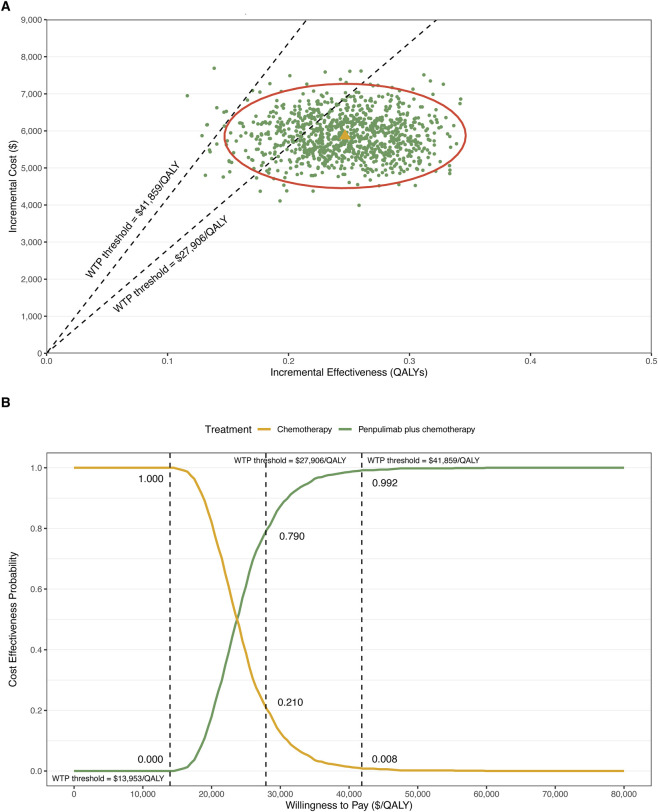
Probabilistic sensitivity analysis. **(A)** The incremental cost-effectiveness scatter plot with 1,000 Monte Carlo iterations; **(B)** The acceptability curves of cost-effectiveness probability at different willingness-to-pay (WTP) values. Abbreviation: QALY, quality-adjusted life year; WTP, willingness-to-pay.

Results from the PSA are shown in Figure 4. Across all simulations, the mean incremental cost of penpulimab plus chemotherapy versus placebo plus chemotherapy was $5,861.41, with a mean incremental QALY gain of 0.25. The probability of cost-effectiveness was 0.0% at the 1× GDP threshold, rising to 79.0% at 2× GDP and 99.2% at 3× GDP.

### 3.3 Price simulation analysis

**FIGURE 5:**
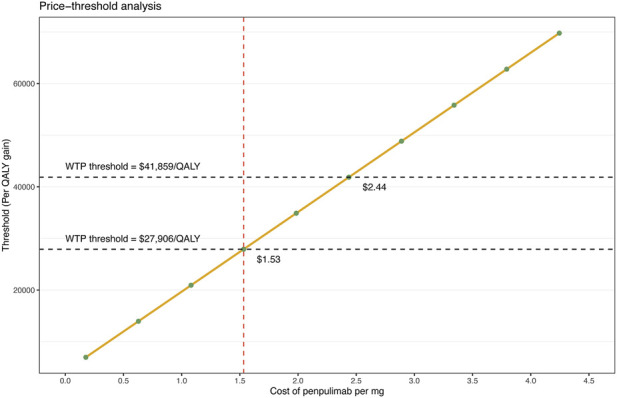
The price simulation of penpulimab. Abbreviation: QALY, quality-adjusted life year; WTP, willingness-to-pay.

Figure 5 illustrates the outcome of the price simulation for penpulimab. With increasing penpulimab price, the ICER rose steadily from $6,976.5/QALY at $0.1761 per mg to $69,765.0/QALY at $4.2453 per mg. Penpulimab plus chemotherapy was estimated to remain cost-effective when penpulimab was priced below $0.6283 per mg at the 1 GDP threshold, below $1.5325 per mg at the 2 GDP threshold, and below $3.3410 per mg at the 3 GDP threshold.

### 3.4 Scenario analyses

The scenario analyses supported the robustness of the base-case results (Table 2). In Scenario 1, with a 0% discount rate, penpulimab plus chemotherapy yielded an ICER of $19,574.62/QALY, with a positive INMB of $2,568.70 and INHB of 0.09 QALYs. In Scenario 2, using a 5% discount rate, the ICER was $24,166.56/QALY, with a positive INMB of $900.80 and INHB of 0.03 QALYs. In Scenario 3, after additionally including low-grade but frequent immune-related AEs, including hypothyroidism and rash, the ICER was $23,922.75/QALY, with a positive INMB of $973.79 and INHB of 0.03 QALYs. All ICERs remained below the WTP threshold of $27,906/QALY.

### 3.5 Subgroup analysis

In the subgroup analysis, penpulimab plus chemotherapy remained a cost-effective treatment strategy in all evaluable subgroups at the threshold of $27,906/QALY (Supplementary Table S6). The ICERs ranged from $6,823.55/QALY in patients with ECOG PS $0 to $13,939.38/QALY in patients with PD-L1-negative disease, and all evaluable subgroups yielded positive INMBs and INHBs. The most economically favorable subgroup was patients with ECOG PS 0. Other subgroups with particularly favorable ICERs included patients with liver metastases, current or former smokers, patients aged ≤50 years, and male patients. By contrast, the least favorable but still cost-effective results were observed in patients with PD-L1-negative disease. Subgroup-specific estimates could not be fully derived for patients treated with carboplatin and those from ex-Asia countries, because corresponding PFS estimates were unavailable.

## 4 Discussion

Penpulimab plus chemotherapy has demonstrated a significant PFS benefit in a recent phase 3 trial, but its economic value in China has been unclear. Given the substantial scale of national healthcare spending, (China Statistical Yearbook, 2025, 2025), this study provides the first evaluation of its economic value for RM-NPC in China. Treatment with penpulimab increased total costs by $5,841.38, yielding gains of 0.29 LYs and 0.25 QALYs, resulting in an ICER of $23,717.68 per QALY. At the WTP threshold of $27,906/QALY, penpulimab plus chemotherapy had a positive INMB of $1,031.53 and a positive INHB of 0.04 QALYs, indicating that first-line penpulimab plus chemotherapy is likely cost-effective compared with placebo plus chemotherapy in China.

ICI–based therapy has reshaped first-line management of RM-NPC. This progress has intensified interest in the economic evaluation of therapeutic alternatives among clinicians, researchers, and healthcare decision-makers. In China, where the burden of NPC remains considerable, selecting the most appropriate treatment requires balancing clinical benefit against economic cost within the local healthcare context. Our base-case ICER indicates that the additional drug costs for penpulimab are justified by the observed survival improvements.

Our findings should be interpreted in the context of the growing economic evidence for first-line immunotherapy combinations in RM-NPC. Although no head-to-head trial has directly compared penpulimab with other PD-1 inhibitors in this setting, published cost-effectiveness studies provide useful indirect contextual evidence. Pei et al. evaluated tislelizumab plus GP as first-line treatment for RM-NPC and reported that it was cost-effective compared with GP alone, with an ICER of $16,859/QALY. (Tang et al., 2024). Han et al. compared toripalimab plus GP and camrelizumab plus GP using data from JUPITER-02 and CAPTAIN-1st, reporting an ICER of $6,696/QALY for toripalimab plus chemotherapy, whereas camrelizumab plus chemotherapy had a substantially higher ICER of $55,305/QALY and was not cost-effective at the prespecified Chinese WTP threshold. (Kun et al., 2022). An updated analysis further compared camrelizumab-, tislelizumab-, and toripalimab-based first-line combinations and suggested that toripalimab was the most economically favorable option among the evaluated regimens, with ICERs of $24,331/QALY, $20,762/QALY, and $18,776/QALY for camrelizumab, tislelizumab, and toripalimab combinations, respectively. (Qiu et al., 2025). In contrast, pembrolizumab, an imported PD-1 inhibitor, produced a much higher ICER of $422,535/QALY versus chemotherapy in RM-NPC, far exceeding commonly used WTP thresholds. (Nie et al., 2024). Toripalimab plus chemotherapy has also been evaluated in the United States, where it remained likely cost-effective but at a higher ICER of $74,004/QALY under a higher local WTP threshold. (Lian et al., 2024). Sintilimab plus chemoradiotherapy was also deemed cost-effective in high-risk locoregionally advanced NPC (ICER $7,819.67 per QALY). (She et al., 2025). Taken together, these studies suggest that the economic value of PD-1 inhibitor–based regimens in NPC varies substantially across agents, clinical settings, countries, drug prices, reimbursement mechanisms, and WTP thresholds. Such discrepancies underscore the impact of local drug pricing and negotiated discounts, with domestic PD-1 antibodies priced more favorably than imported drugs. Comparable findings have also been observed beyond NPC. A recent network meta-analysis in recurrent or metastatic head and neck squamous cell carcinoma showed finotonlimab plus chemotherapy to be the most cost-effective option, with an ICER of $161.13 per QALY versus cetuximab plus chemotherapy. (Liu et al., 2025). In contrast, pembrolizumab-containing regimens were markedly less cost-effective, with ICERs of $85,132/QALY for pembrolizumab alone and $203,545/QALY for pembrolizumab combined with chemotherapy. (Liu et al., 2025). Penpulimab plus anlotinib is similarly cost-effective as first-line therapy for unresectable hepatocellular carcinoma in China (ICER $34,862.65/QALY). (JinDi et al., 2026). In metastatic squamous non-small-cell lung cancer, penpulimab plus chemotherapy was also cost-effective versus carboplatin-paclitaxel (ICER $14,918.81 per QALY). (Wang et al., 2025). Our analysis adds penpulimab-specific evidence to this landscape and indicates that penpulimab plus chemotherapy is economically favorable compared with placebo plus chemotherapy under current Chinese pricing. However, because these comparisons are based on separate trials and published economic models with differences in modeling assumptions, and cost inputs, they should be regarded as contextual rather than definitive indirect comparisons. Future real-world comparative analyses will be needed to define the relative economic value of penpulimab more precisely versus other first-line immunotherapy combinations, including toripalimab, tislelizumab, camrelizumab, and sintilimab-based regimens. The findings of our study were generally robust. One-way sensitivity analysis identified the utility value of the PFS state as the most influential parameter, followed by the price of penpulimab, discount rate, and utility value of the PD state. All other parameters exerted relatively minor effects on the model outcomes. Notably, only substantial variation in the PFS utility value increased the ICER beyond the WTP threshold, highlighting that the cost-effectiveness of penpulimab plus chemotherapy is largely driven by the quality-of-life benefits associated with prolonged PFS. Findings from the PSA reinforced the robustness of the result. At a WTP threshold of two times the Chinese GDP *per capita*, penpulimab plus chemotherapy was cost-effective in 79.0% of simulations, and this probability increased to 99.2% at three times GDP *per capita*. These results indicate a high likelihood that penpulimab plus chemotherapy remains a cost-effective first-line treatment option for patients with RM-NPC across a wide range of plausible parameter uncertainties.

The price simulation analysis is among the most policy-relevant findings of this study. In the underlying clinical evidence, penpulimab plus chemotherapy improved PFS relative to placebo plus chemotherapy. Longer survival also implies a longer duration of immunotherapy exposure and additional downstream medical spending, which increases total cost. By contrast, chemotherapy alone remains financially competitive at lower or moderate WTP levels because its total cost is substantially lower. This interaction between clinical benefit and acquisition cost is clearly reflected in the simulation. The ICER increased from $6,976.5/QALY at a price of $0.1761 per mg to $69,765.0/QALY at $4.2453 per mg, and penpulimab remained cost-effective only below $0.6283, $1.5325, and $3.3410 per mg per mg at the 1×, 2×, and 3× GDP thresholds, respectively. These results align with the sensitivity analyses, in which penpulimab price emerged as a major value driver. They are also highly relevant to China, where PD-1 inhibitor prices and overall treatment costs can vary over time and across settings because of national reimbursement negotiations, procurement mechanisms, and institutional differences. Moreover, regional economic heterogeneity is substantial. According to 2024 official statistics, per-capita GDP was $ 31,956 in Beijing, $ 30,412 in Shanghai, $ 18,987 in Zhejiang, $ 13,666 in Shandong, $ 10,625 in Jiangxi, and $ 7,398 in Gansu. (China Statistical Yearbook, 2025, 2025). Thus, the economic attractiveness of penpulimab is not fixed. It depends on the interaction between clinical benefit, local WTP, and drug acquisition cost. In practice, these thresholds may inform reimbursement negotiation, hospital procurement, inclusion in basic medical insurance, and broader access strategies, making this type of analysis especially useful for dynamic pricing and formulary decision-making in oncology systems under budget pressure.

The subgroup analysis provides useful insight into heterogeneity in economic value. The main message is that penpulimab plus chemotherapy remained cost-effective across all evaluable subgroups. Patients with ECOG PS 0 showed the most favorable economic outcomes, followed by those with liver metastases, current/former smokers, age≤50, males, and PD-L1–positive disease. Several biomarkers and clinical characteristics may partly explain the observed heterogeneity. PD-L1 expression is a candidate predictive biomarker in NPC, and plasma EBV-DNA is a well-established prognostic marker. (Chen et al., 2021; Hsu et al., 2017; Yip et al., 2014). Therefore, subgroups deriving greater relative clinical benefit may also show greater economic value. (Chen et al., 2021; Hsu et al., 2017). However, because subgroup-specific Kaplan–Meier curves and patient-level data were unavailable, these subgroup findings should be interpreted as exploratory rather than confirmatory.

Several limitations should be acknowledged. First, long-term survival was extrapolated beyond the available trial follow-up, which inevitably introduces uncertainty. To reduce this uncertainty, we reconstructed individual patient data from the published Kaplan–Meier curves and evaluated a broad range of conventional and flexible survival models. Nevertheless, mature OS data or real-world long-term follow-up may alter the projected survival benefits and should be incorporated in future updates. Second, health utility values were sourced from published literature, rather than directly measured in the pivotal penpulimab trial. To mitigate this uncertainty, we varied the utility values over predefined ranges in deterministic sensitivity analyses, assigned beta distributions in PSA, and further assessed the joint effect of PFS utility and penpulimab cost in a two-way sensitivity analysis. Future studies incorporating trial-based or real-world EQ-5D measurements in Chinese patients with RM-NPC would further improve the precision of economic evaluations. Third, although drug acquisition costs were based on median prices from Yaozhi to improve generalizability beyond a single-hospital setting, actual prices may still vary across provinces, hospital levels, procurement platforms, reimbursement policies, and future national price negotiations. Fourth, our model compared penpulimab plus chemotherapy only with chemotherapy alone and did not directly compare penpulimab with other first-line immunotherapy regimens, such as toripalimab, camrelizumab, or tislelizumab plus chemotherapy. Fifth, the subgroup analyses were based on trial-reported HRs rather than subgroup-specific Kaplan–Meier curves or patient-level survival data. However, applying subgroup HRs required an additional proportional hazards approximation. Although this approach is commonly used when only subgroup HRs are available, it may not fully capture time-varying treatment effects. Future studies using subgroup-specific survival curves, patient-level data, or mature real-world evidence are needed to validate these findings. Finally, we adopted a Chinese healthcare payer perspective, and results may not generalize to countries with different costs or WTP thresholds. Future analyses incorporating longer trial follow-up, mature OS data, and real-world evidence would further strengthen the evidence base for reimbursement and clinical decision-making.

## 5 Conclusion

In conclusion, first-line penpulimab plus chemotherapy represents a cost-effective strategy for RM-NPC in China, achieving survival extension at an acceptable incremental cost. Given the substantial clinical and societal burden of RM-NPC, economically sustainable innovation is essential. These findings may inform reimbursement and treatment decisions by indicating that, under current pricing, penpulimab plus chemotherapy is a cost-effective option. Nevertheless, continued evidence generation is warranted to confirm its long-term value and to optimize access in Chinese healthcare.

## Data Availability

The original contributions presented in the study are included in the article/Supplementary Material, further inquiries can be directed to the corresponding author.
