# Supplementary material for: Cost-effectiveness analysis of first-line penpulimab plus chemotherapy for recurrent or metastatic nasopharyngeal carcinoma in China
Source: Front Pharmacol. 2026 Jul 3;17:1885531. doi: 10.3389/fphar.2026.1885531 (PMC13375980; doi:10.3389/fphar.2026.1885531)
Supplement: Supplementary file 1 [file DataSheet1.pdf]

Supplementary File:

Cost-effectiveness analysis of first-line penpulimab plus chemotherapy for recurrent or metastatic nasopharyngeal carcinoma in China

Index

Index 1
Table S1. CHEERS 2022 Checklist. 2
Table S2. Parametric survival distributions evaluated for OS and PFS. 5
Table S3. Comparison of median overall survival and median progression-free survival between the fitted curve and the reported curve. 7
Table S4. Incidence and management costs of low-grade but frequent immune-related adverse events included in Scenario 3. 9
Table S5. Subsequent treatment regimen, proportion, and duration. 8
Table S6. Results of subgroup analyses. 10
Figure S1. KM curves for OS (A) and PFS (B). 12
Figure S2. Exponential Kaplan-Meier curves of overall survival in penpulimab plus chemotherapy. 13
Figure S3. Exponential Kaplan-Meier curves of progression-free survival in penpulimab plus chemotherapy. 14
Figure S4. Exponential Kaplan-Meier curves curves of overall survival in chemotherapy. 15
Figure S5. Exponential Kaplan-Meier curves curves of progression-free survival in chemotherapy. 16
Reference 17

Table S1. CHEERS 2022 Checklist.

Topic	No.	Item	Reported
**Title**	1	Identify the study as an economic evaluation and specify the interventions being compared.	Yes
**Abstract**	2	Provide a structured summary that highlights context, key methods, results, and alternative analyses.	Yes
**Introduction**			
**Background and objectives**	3	Give the context for the study, the study question, and its practical relevance for decision making in policy or practice.	Yes
**Methods**			
**Health economic analysis plan**	4	Indicate whether a health economic analysis plan was developed and where available.	Not applicable
**Study population**	5	Describe characteristics of the study population (such as age range, demographics, socioeconomic, or clinical characteristics).	Yes
**Setting and location**	6	Provide relevant contextual information that may influence findings.	Yes
**Comparators**	7	Describe the interventions or strategies being compared and why chosen.	Yes
**Perspective**	8	State the perspective(s) adopted by the study and why chosen.	Yes
**Time horizon**	9	State the time horizon for the study and why appropriate.	Yes
**Discount rate**	10	Report the discount rate(s) and reason chosen.	Yes
**Selection of outcomes**	11	Describe what outcomes were used as the measure(s) of benefit(s) and harm(s).	Yes
**Measurement of outcomes**	12	Describe how outcomes used to capture benefit(s) and harm(s) were measured.	Yes
**Valuation of outcomes**	13	Describe the population and methods used to measure and value outcomes.	Yes
**Measurement and valuation of resources and costs**	14	Describe how costs were valued.	Yes
**Currency, price date, and conversion**	15	Report the dates of the estimated resource quantities and unit costs, plus the currency and year of conversion.	Yes

Topic	No.	Item	Reported
**Rationale and description of model**	16	If modelling is used, describe in detail and why used. Report if the model is publicly available and where it can be accessed.	Yes
**Analytics and assumptions**	17	Describe any methods for analysing or statistically transforming data, any extrapolation methods, and approaches for validating any model used.	Yes
**Characterising heterogeneity**	18	Describe any methods used for estimating how the results of the study vary for subgroups.	Yes
**Characterising distributional effects**	19	Describe how impacts are distributed across different individuals or adjustments made to reflect priority populations.	Not applicable
**Characterising uncertainty**	20	Describe methods to characterise any sources of uncertainty in the analysis.	Yes
**Approach to engagement with patients and others affected by the study**	21	Describe any approaches to engage patients or service recipients, the general public, communities, or stakeholders (such as clinicians or payers) in the design of the study.	Not applicable
**Results**			
**Study parameters**	22	Report all analytic inputs (such as values, ranges, references) including uncertainty or distributional assumptions.	Yes
**Summary of main results**	23	Report the mean values for the main categories of costs and outcomes of interest and summarise them in the most appropriate overall measure.	Yes
**Effect of uncertainty**	24	Describe how uncertainty about analytic judgments, inputs, or projections affect findings. Report the effect of choice of discount rate and time horizon, if applicable.	Yes
**Effect of engagement with patients and others affected by the study**	25	Report on any difference patient/service recipient, general public, community, or stakeholder involvement made to the approach or findings of the study	Not applicable
**Discussion**			
**Study findings, limitations, generalisability, and current knowledge**	26	Report key findings, limitations, ethical or equity considerations not captured, and how these could affect patients, policy, or practice.	Yes
**Other relevant information**			

Topic	No.	Item	Reported
**Source of funding**	27	Describe how the study was funded and any role of the funder in the identification, design, conduct, and reporting of the analysis	Yes
**Conflicts of interest**	28	Report authors conflicts of interest according to journal or International Committee of Medical Journal Editors requirements.	Yes

Table S2. Parametric survival distributions evaluated for OS and PFS.

Model	OS for penpulimab plus chemotherapy			PFS for penpulimab plus chemotherapy			OS for chemotherapy			PFS for chemotherapy		
	**LnL**	**Params**	**AIC**	**LnL**	**Params**	**AIC**	**LnL**	**Params**	**AIC**	**LnL**	**Params**	**AIC**
Exponential	-61.3659	1	124.7319	-76.1788	1.0000	154.3576	-61.3463	1	124.6926	-100.0861	1	202.1722
Weibull	-55.2398	2	114.4796	-72.5481	2.0000	149.0963	-52.7552	2	109.5104	-78.4926	2	160.9852
Gamma	-54.9508	2	113.9017	-70.4875	2.0000	144.9749	-51.7779	2	107.5557	-69.9132	2	143.8264
Log-normal	-55.5823	2	115.1647	-64.6533	2.0000	133.3065	-50.3026	2	104.6053	-63.5391	2	131.0781
Gompertz	-57.4161	2	118.8322	-76.0329	2.0000	156.0657	-56.4506	2	116.9012	-94.6666	2	193.3333
Log-logistic	**-54.7220**	**2**	**113.4440**	-65.9747	2.0000	135.9495	-51.6921	2	107.3843	-59.3204	2	122.6409
Generalized Gamma	-54.9161	3	115.8322	-63.9027	3.0000	133.8055	-49.6117	3	105.2234	-63.4538	3	132.9077
FP1-1	-60.2227	2	124.4455	-70.2518	2.0000	144.5036	-59.9743	2	123.9487	-70.6213	2	145.2426
FP1-2	-55.1331	2	114.2662	-68.1963	2.0000	140.3926	-51.2150	2	106.4300	-71.1381	2	146.2762
FP2-1	-54.7040	3	115.4080	-61.4334	4.0000	130.8669	-50.1388	3	106.2776	-56.8587	3	119.7174
FP2-2	-54.2651	3	114.5302	-64.0002	3.0000	134.0003	-50.4135	3	106.8269	-56.9834	3	119.9667
RCS1	-54.0883	3	114.1766	-68.9357	3.0000	143.8713	-52.1958	3	110.3916	-51.6705	6	115.3411

Model	OS for penpulimab plus chemotherapy			PFS for penpulimab plus chemotherapy			OS for chemotherapy			PFS for chemotherapy		
	**LnL**	**Params**	**AIC**	**LnL**	**Params**	**AIC**	**LnL**	**Params**	**AIC**	**LnL**	**Params**	**AIC**
RCS2	-53.6086	4	115.2172	**-58.4956**	**4.0000**	**124.9911**	-51.7181	4	111.4361	-53.5888	5	117.1775
RP-hazard-1	-55.2398	2	114.4796	-60.1649	3.0000	126.3298	-50.3340	3	106.6680	-51.9957	6	115.9913
RP-hazard-2	-53.6208	4	115.2417	-58.3162	5.0000	126.6325	-48.6730	5	107.3460	-53.2409	4	114.4817
RP-odds-1	**-54.7220**	**2**	**113.4440**	-60.4699	3.0000	126.9399	-50.2704	3	106.5408	-52.2142	6	116.4284
RP-odds-2	-53.6159	4	115.2318	-58.4552	5.0000	126.9104	-51.6921	2	107.3843	**-53.0377**	**4**	**114.0754**
RP-normal-1	-55.5823	2	115.1646	-58.3434	5.0000	126.6868	**-50.3026**	**2**	**104.6053**	-52.2691	6	116.5382
RP-normal-2	-53.6138	4	115.2277	-59.2598	4.0000	126.5196	-49.8412	3	105.6825	-53.0968	4	114.1936

Abbreviation: AIC = Akaike information criterion. BIC = Bayesian information criterion. OS = overall survival. PFS = progression-free survival.

Table S3. Comparison of median overall survival and median progression-free survival between the fitted curve and the reported curve.

Parameter	Baseline value	Minimum	Maximum	Reference
The original mOS of the penpulimab plus chemotherapy group (months)	NR	25.00	NE	Model Fitting
The fitting mOS of the penpulimab plus chemotherapy group (months)	NR	25.1	NE	Model Fitting
The original mPFS of the penpulimab plus chemotherapy group (months)	9.63	7.13	12.45	Model Fitting
The fitting mPFS of the penpulimab plus chemotherapy group (months)	9.66	7.14	12.55	Model Fitting
The original mOS of the chemotherapy group (months)	NR	24.00	NE	Model Fitting
The fitting mOS of the chemotherapy group (months)	NR	24.8	NE	Model Fitting
The original mPFS of the chemotherapy group (months)	7.00	6.90	7.29	Model Fitting
The fitting mPFS of the chemotherapy group (months)	7.00	6.90	8.11	Model Fitting

Abbreviations: OS, overall survival; PFS, progression-free survival

Table S4. Subsequent treatment regimen, proportion, and duration.

Group	Drug/Treatment	Dosage	Frequency of administration	Duration	Proportion	References
Chemotherapy group	Docetaxel	$75 mg/m^2$	Every three weeks	4.0 months	45.58%	[1, 2]
	Camrelizumab	200 mg	Every three weeks	5.1 months	28.57%	[2, 3]
	Anlotinib	12 mg	Day 1 to day 14, oral daily, every three weeks	3.0 months	15.65%	[2, 4]
	Crossover to penpulimab	200 mg	Every three weeks	4.1 months	46.26%	[2, 5]
Penpulimab plus chemotherapy group	Docetaxel	$75 mg/m^2$	Every three weeks	4.0 months	43.75%	[1, 2]
	Camrelizumab	200 mg	Every three weeks	5.1 months	24.31%	[2, 3]
	Anlotinib	12 mg	Day 1 to day 14, oral daily, every three weeks	3.0 months	11.81%	[2, 4]
	Crossover to penpulimab	200 mg	Every three weeks	4.1 months	NA	[2, 5]

Table S5. Incidence and management costs of low-grade but frequent immune-related adverse events included in Scenario 3.

irAE	Cost components	Incidence	Cost, USD	References
Hypothyroidism	Oncology/dermatology/endocrinology outpatient visit; venous blood sampling; thyroid function tests; thyroid ultrasound; levothyroxine sodium	17.8%[2]	$34.37	Zhejiang Provincial catalog*[6]
Rash	Oncology/dermatology outpatient visit; venous blood sampling; complete blood count; antihistamines, topical corticosteroids, emollients, or other symptomatic treatment	7.5%[2]	$12.02	Zhejiang Provincial catalog*[6]

* Costs sourced from the Zhejiang Provincial Basic Medical Insurance Service Item Catalogue, considered representative of national pricing.

Table S6. Results of subgroup analyses.

Subgroup	OS HR (95% CI)	PFS HR (95% CI)	ICER ($/LY)	ICER ($/QALY)	INMB ($)	INHB (QALY)
**Age**						
<=50	0.51 (0.38, 0.69)	0.59 (0.39, 0.9)	5,209.12	7,898.69	30,908.12	1.11
>50	0.55 (0.29, 1.03)	0.46 (0.30, 0.71)	7,532.54	10,102.96	27,981.29	1.00
**Sex**						
Male	0.50 (0.38, 0.66)	0.57 (0.41, 0.79)	5,309.14	7,983.90	32,020.05	1.15
Female	0.47 (0.21, 1.04)	0.44 (0.22, 0.89)	6,617.66	9,173.28	35,929.74	1.29
**Stage of disease at study entry**						
Primary Metastases	0.49 (0.36,0.66)	0.53 (0.32, 0.87)	5,650.39	8,309.92	33,220.83	1.19
Recurrent	0.54 (0.30, 0.97)	0.51 (0.35, 0.73)	6,604.46	9,306.01	28,257.32	1.01
**ECOG PS**						
0	0.47 (0.31, 0.69)	0.65 (0.40, 1.06)	4,295.68	6,823.55	35,184.19	1.26
1	0.53 (0.37, 0.75)	0.42 (0.28, 0.61)	7,845.39	10,291.51	30,549.55	1.09
**Liver metastases**						
Present	0.37 (0.24, 0.57)	0.5 (0.32, 0.79)	4,714.77	7,170.08	48,617.73	1.74
Absent	0.60 (0.43, 0.85)	0.54 (0.36, 0.80)	7,121.48	9,914.28	22,863.67	0.82
**Investigator's choice of chemotherapy**						
Carboplatin	0.37 (0.24, 0.57)	NA	NA	NA	NA	NA
Cisplatin	0.6 (0.43, 0.85)	0.53 (0.39, 0.71)	7,274.52	10,040.39	22,964.20	0.82

Subgroup	OS HR (95% CI)	PFS HR (95% CI)	ICER ($/LY)	ICER ($/QALY)	INMB ($)	INHB (QALY)
**Smoking status**						
Never	0.56 (0.31, 1.01)	0.46 (0.3, 0.71)	7,697.88	10,257.06	27,161.21	0.97
Current/Former	0.49 (0.36, 0.65)	0.60 (0.40, 0.90)	4,897.48	7,531.16	33,034.33	1.18
**PD-L1 expression level**						
Positive (TPS >=50%)	0.45 (0.33, 0.61)	0.50 (0.31,0.81)	5,532.60	8,125.87	37,927.44	1.36
Negative (TPS <50%or NA)	0.78 (0.44, 1.38)	0.55 (0.38, 0.81)	11,606.61	13,939.38	10,557.39	0.38
**EBV-DNA level**						
<500 copies/mL	0.55 (0.38, 0.79)	0.61 (0.36, 1.02)	5,465.73	8,247.49	26,852.58	0.96
>=500 copies/mL	0.43 (0.29, 0.63)	0.45 (0.31, 0.65)	5,928.02	8,479.24	40,604.55	1.46
**Region**						
Asian counties	0.48 (0.37, 0.64)	0.52 (0.39, 0.70)	5,649.13	8,290.80	34,364.73	1.23
ex-Asia countries	0.29 (0.07, 1.24)	NA	NA	NA	NA	NA

Abbreviation: OS, overall survival; PFS, progression-free survival; HR, hazard ratio; CI, confidence interval; LY, life-year; QALY, quality-adjusted life year; ICER, incremental cost-effectiveness ratio; INMB, incremental net monetary benefits; INHB, incremental net health benefits; WTP, willingness-to-pay; ECOG PS, Eastern Cooperative Oncology Group performance status.

Figure S1. KM curves for OS (A) and PFS (B).

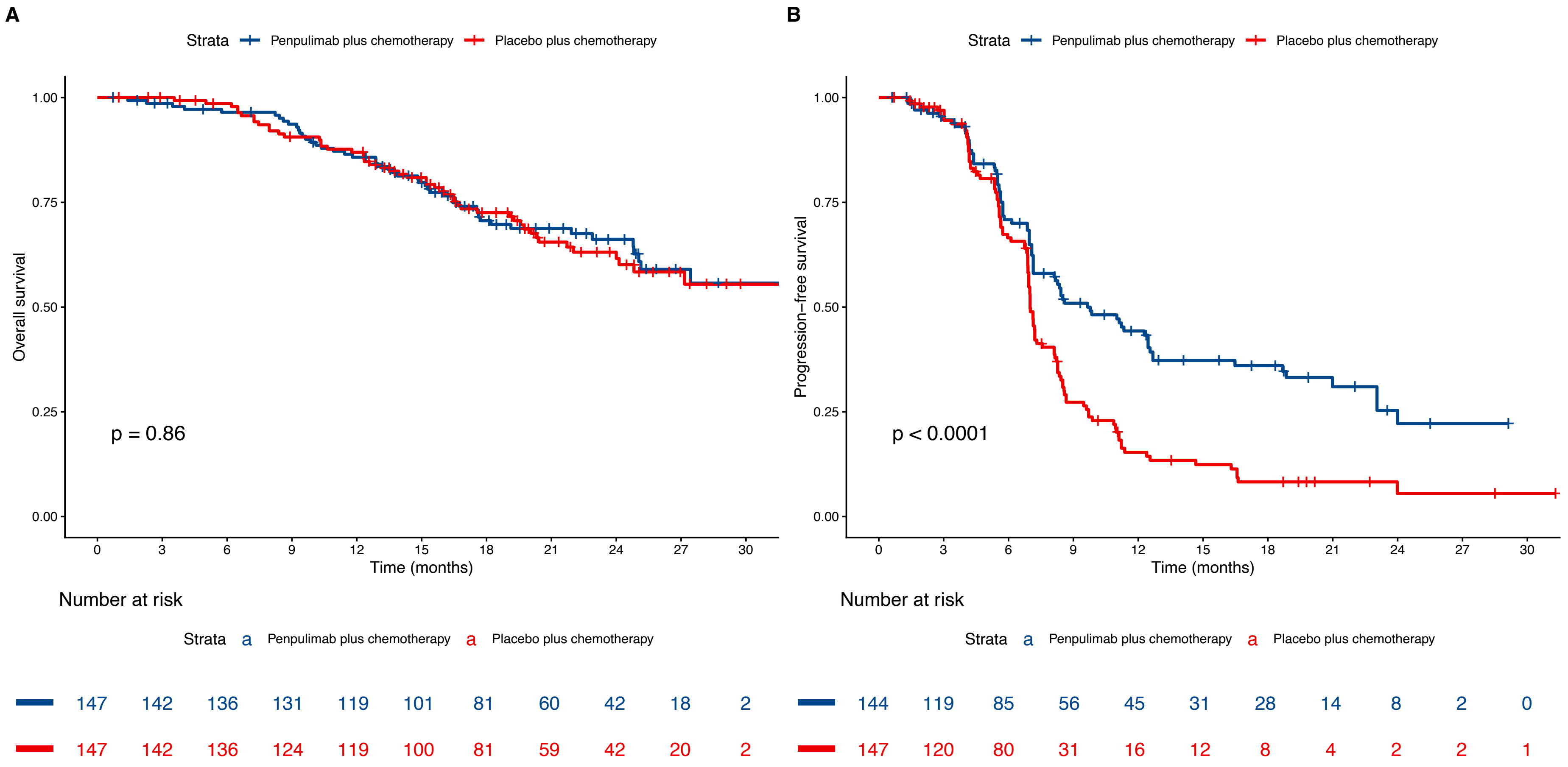

Abbreviation: KM curves = Kaplan-Meier curves. OS = overall survival. PFS = progression-free survival.

Figure S2. Exponential Kaplan-Meier curves of overall survival in penpulimab plus chemotherapy.

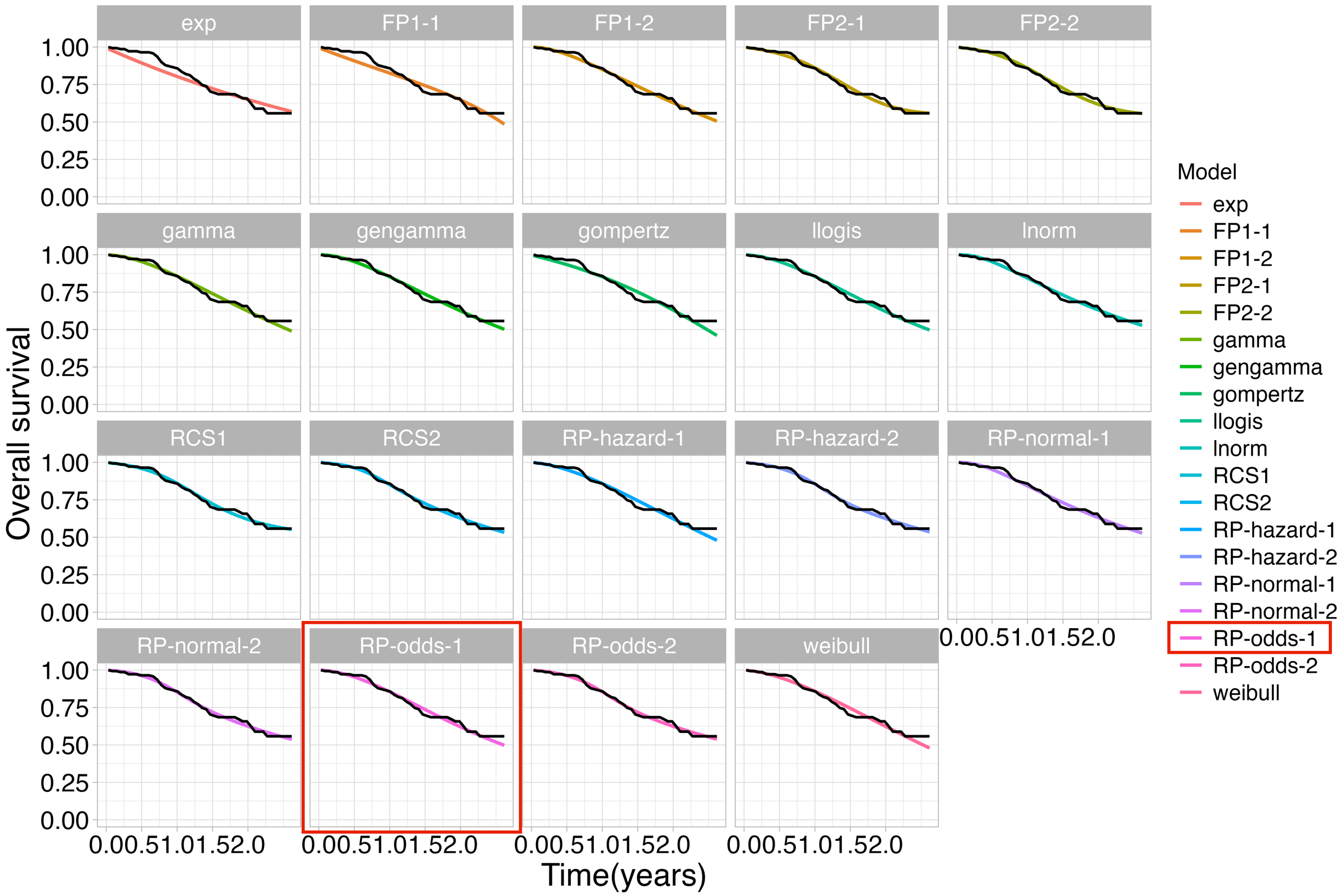

Figure S3. Exponential Kaplan-Meier curves of progression-free survival in penpulimab plus chemotherapy.

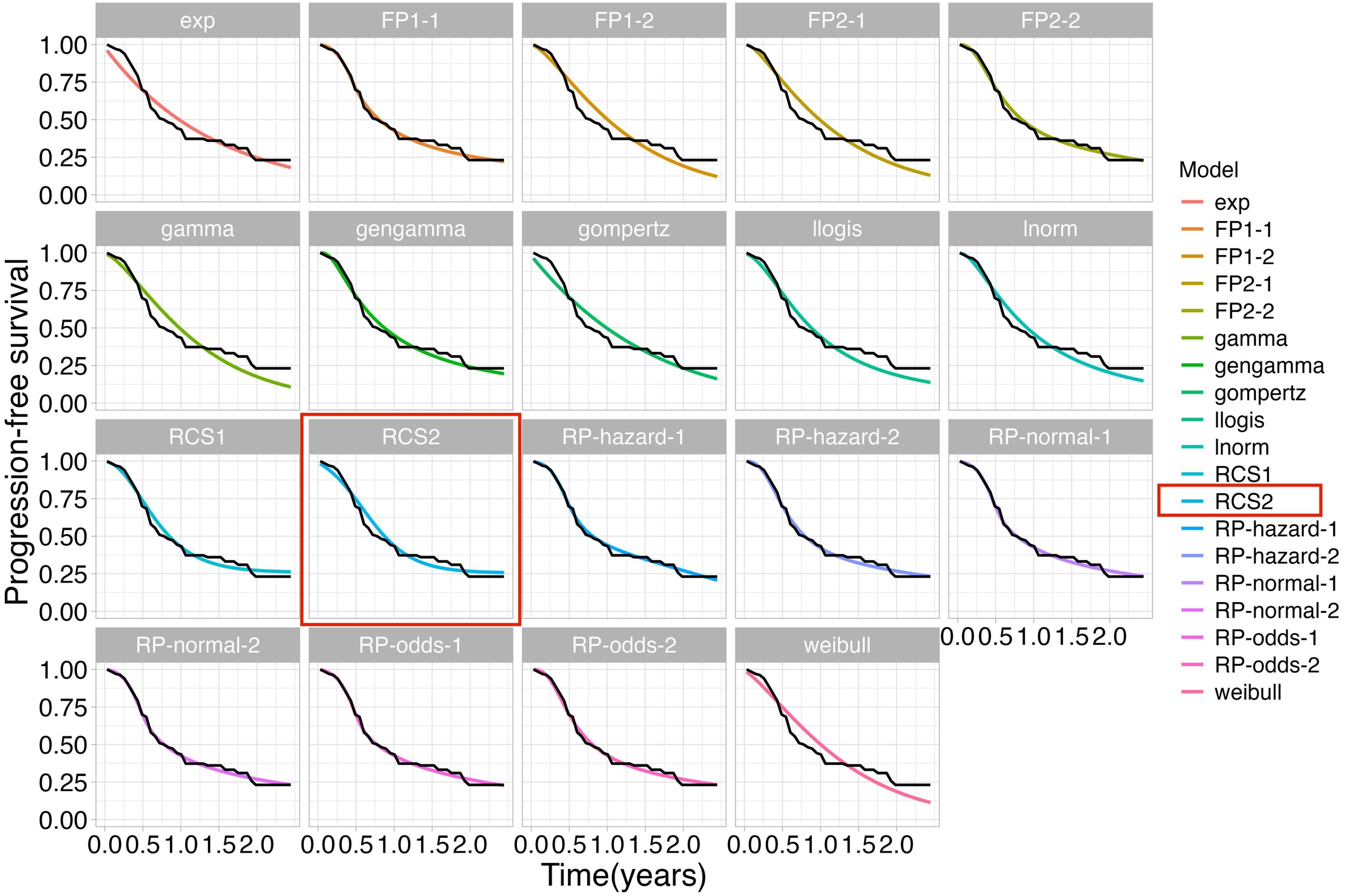

Figure S4. Exponential Kaplan-Meier curves curves of overall survival in chemotherapy.

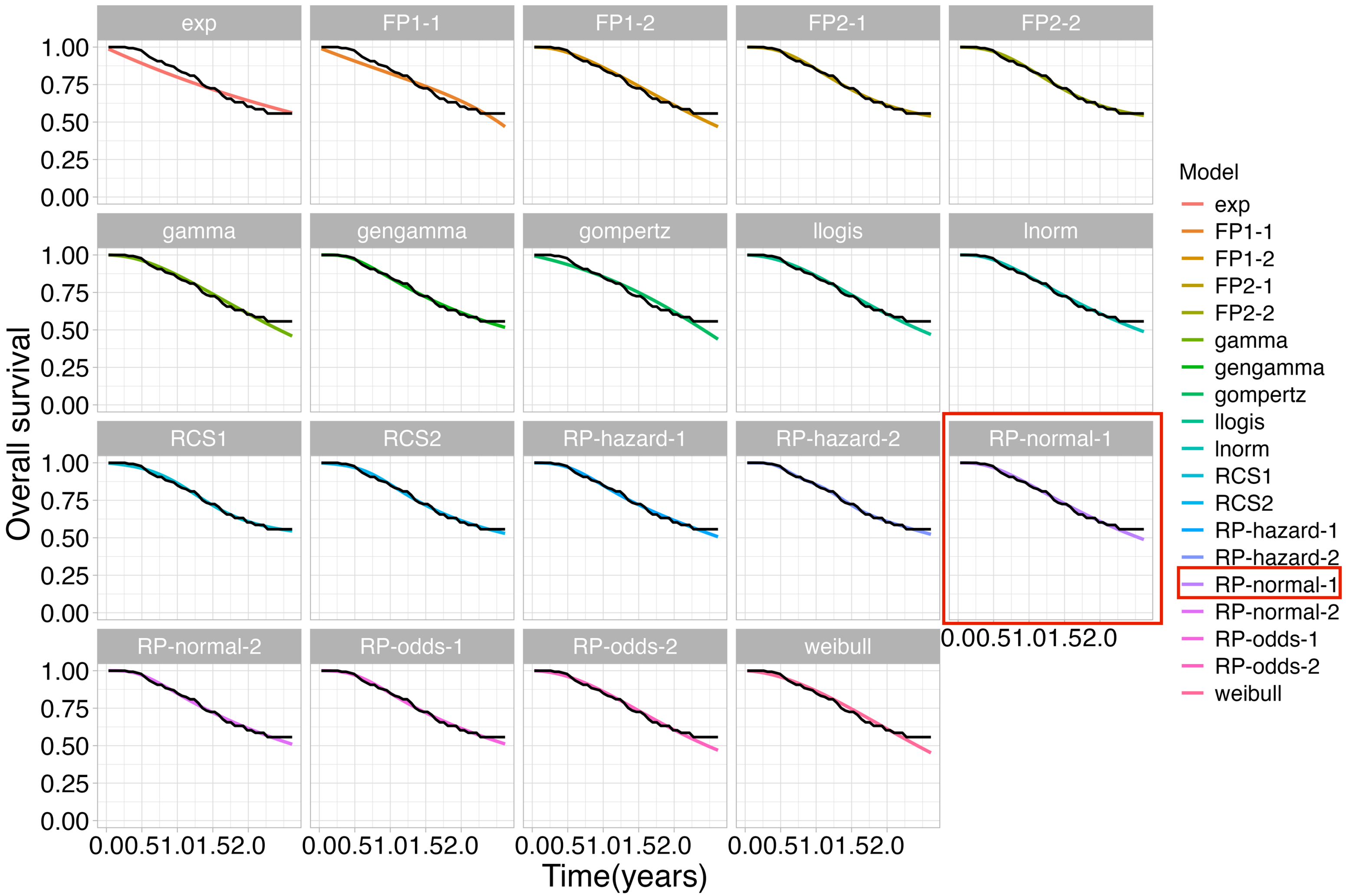

Figure S5. Exponential Kaplan-Meier curves curves of progression-free survival in chemotherapy.

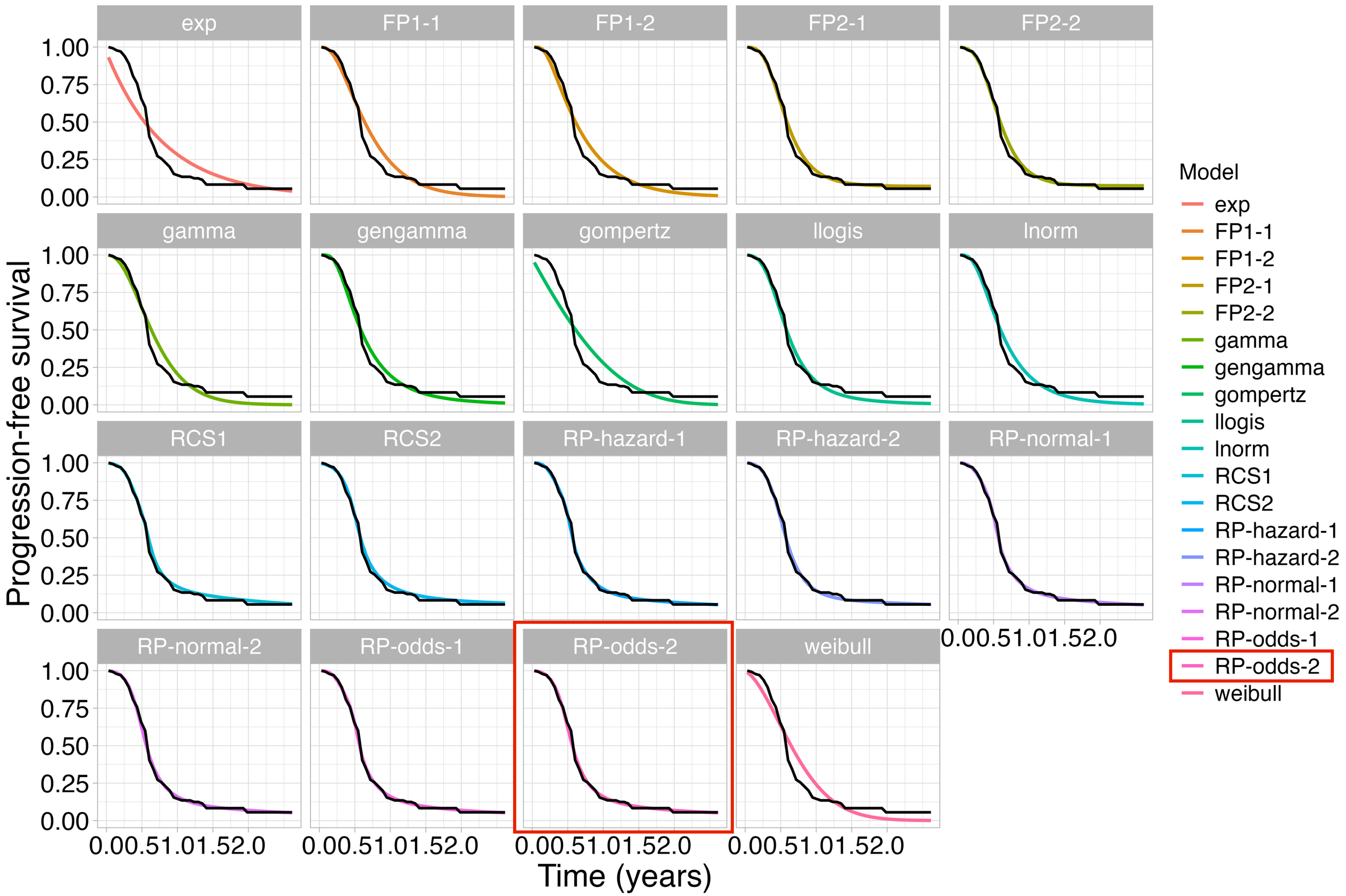

Reference

1. Ngeow J, Lim WT, Leong SS, Ang MK, Toh CK, Gao F, et al. Docetaxel is effective in heavily pretreated patients with disseminated nasopharyngeal carcinoma. Ann Oncol. 2011;22(3):718-22. https://doi.org/10.1093/annonc/mdq425 PMID: 20716628
2. Huang S, Liu F, Qu S, Chen L, Zhou P, Qu S, et al. Anti-PD-1 antibody penpulimab plus chemotherapy for recurrent or metastatic nasopharyngeal carcinoma: a randomized, double-blind phase 3 study. Signal Transduct Target Ther. 2026;11(1). https://doi.org/10.1038/s41392-026-02645-0 PMID: 41946687
3. Fang W, Yang Y, Ma Y, Hong S, Lin L, He X, et al. Camrelizumab (SHR-1210) alone or in combination with gemcitabine plus cisplatin for nasopharyngeal carcinoma: results from two single-arm, phase 1 trials. Lancet Oncol. 2018;19(10):1338-50. https://doi.org/10.1016/s1470-2045(18)30495-9 PMID: 30213452
4. Fang Y, Su N, Zou Q, Cao Y, Xia Y, Tang L, et al. Anlotinib as a third-line or further treatment for recurrent or metastatic nasopharyngeal carcinoma: a single-arm, phase 2 clinical trial. BMC Med. 2023;21(1):423. https://doi.org/10.1186/s12916-023-03140-x PMID: 37936166
5. Chen X, Wang W, Zou Q, Zhu X, Lin Q, Jiang Y, et al. Penpulimab, an anti-PD-1 antibody, for heavily pretreated metastatic nasopharyngeal carcinoma: a single-arm phase II study. Signal Transduct Target Ther. 2024;9(1):148. https://doi.org/10.1038/s41392-024-01865-6 PMID: 38890298
6. Jin L, Han L, Zeng J. Cost-effectiveness analysis based on two models of sacituzumab tirumotecan versus platinum-based chemotherapy as second-line treatment in EGFR-mutant non-small-cell lung cancer. Front Public Health. 2026;14:1802471. https://doi.org/10.3389/fpubh.2026.1802471 PMID: 42180459
